# Changes in cognitive and behavioral control after lamotrigine and intensive dialectical behavioral therapy for severe, multi-impulsive bulimia nervosa: an fMRI case study

**DOI:** 10.1007/s40519-021-01308-z

**Published:** 2021-10-18

**Authors:** Laura A. Berner, Erin E. Reilly, Xinze Yu, Angeline Krueger, Mary Ellen Trunko, Leslie K. Anderson, Joanna Chen, Alan N. Simmons, Walter H. Kaye

**Affiliations:** 1 Department of Psychiatry, University of California San Diego, San Diego, CA USA; 2 Present Address: Department of Psychiatry, Icahn School of Medicine at Mount Sinai, New York, NY USA; 3 Present Address: Department of Psychology, Hofstra University, Hempstead, NY USA; 4 Present Address: Department of Psychology, Drexel University, Philadelphia, PA USA; 5 VA San Diego Healthcare System, San Diego, CA USA

**Keywords:** Binge eating, Purging, Bulimia nervosa, Cognitive control, Dialectical behavior therapy, Lamotrigine

## Abstract

**Purpose:**

Adults with bulimia nervosa (BN) and co-occurring emotional dysregulation and multiple impulsive behaviors are less responsive to existing interventions. Initial data suggest that the combination of Dialectical Behavior Therapy (DBT) and a mood stabilizer, lamotrigine, significantly reduces symptoms of affective and behavioral dysregulation in these patients. Identifying candidate neurobiological mechanisms of change for this novel treatment combination may help guide future randomized controlled trials and inform new and targeted treatment development. Here, we examined neurocognitive and symptom changes in a female patient with BN and severe affective and behavioral dysregulation who received DBT and lamotrigine.

**Methods:**

Go/no-go task performance data and resting-state functional MRI scans were acquired before the initiation of lamotrigine (after 6 weeks in an intensive DBT program), and again after reaching and maintaining a stable dose of lamotrigine. The patient completed a battery of symptom measures biweekly for 18 weeks over the course of treatment.

**Results:**

After lamotrigine initiation, the patient made fewer errors on a response inhibition task and showed increased and new connectivity within frontoparietal and frontolimbic networks involved in behavioral and affective control. Accompanying this symptom improvement, the patient reported marked reductions in bulimic symptoms, behavioral dysregulation, and reactivity to negative affect, along with increases in DBT skills use.

**Conclusion:**

Improved response inhibition and cognitive control network connectivity should be further investigated as neurocognitive mechanisms of change with combined DBT and lamotrigine for eating disorders. Longitudinal, controlled trials integrating neuroimaging and symptom measures are needed to fully evaluate the effects of this treatment.

**Level of Evidence:**

IV: Evidence obtained from multiple time series with or without the intervention, such as case studies.

**Supplementary Information:**

The online version contains supplementary material available at 10.1007/s40519-021-01308-z.

## Introduction

Bulimia nervosa (BN), characterized in part by regular binge eating and purging, is associated with medical complications, functional impairment, and high rates of mortality and chronicity [1–7]. First-line pharmacotherapy (selective serotonin reuptake inhibitors) and psychotherapy (cognitive behavioral therapy) promote full remission in less than half of patients with BN [8, 9]. Moreover, BN frequently co-occurs with high levels of affective dysregulation and non-eating-related impulsive behaviors, such as shoplifting and substance use [10–12]. Some data indicate that the subgroup of individuals with BN who engage in multiple other impulsive behaviors, which range from 3% of community samples to 44% of mixed clinical and community samples [13–15], show a consistently poorer response to existing eating disorder treatments [9, 13, 16, 17]. However, little is known about the neurobiological mechanisms that promote this more severe and treatment-resistant variant of BN. Identifying neurobiological targets for innovative treatment approaches within this severely dysregulated population is imperative.

Mounting evidence has highlighted a potential role for deficits in cognitive and behavioral control in BN symptoms. Self-report data suggest that binge eating and purging may represent impulsive attempts to regulate affect in the context of poor affect regulation abilities [18]. In support of this notion, affective dysregulation is linked to greater behavioral impulsivity in BN [19], and individuals with BN exhibit generalized difficulties with attentional and motor control [10, 20, 21]. There have been relatively few neuroimaging studies focused on BN, but existing findings suggest that more frequent binge eating and purging is associated with more pronounced functional and anatomical abnormalities in frontostriatal, frontoparietal, and limbic regions [21–25], further suggesting that alterations in brain circuits that govern self-regulatory control may contribute to BN.

Given research implicating self-regulatory control in BN, there has been increasing interest in adapting treatments designed to target affective lability and behavioral disinhibition for use in this population. For example, Dialectical Behavior Therapy (DBT), originally developed for chronically suicidal individuals with borderline personality disorder BPD; [26, 27] has shown promising initial effects in individuals with bulimic-spectrum eating disorders [28, 29]. DBT combines individual therapy, weekly skills groups, phone coaching, and a therapist consultation group [26]. The treatment outlines a structured framework for therapists to target emotion dysregulation and impulsive behavior. However, adjunctive treatment may be indicated for individuals who struggle to engage in intensive psychological treatment because of impulsive behaviors (e.g., leaving treatment prematurely). Our group has published two recent case series indicating that the addition of a mood stabilizer, lamotrigine, to DBT may be particularly helpful for patients with binge/purge eating disorders and significant affective and behavioral dysregulation [30, 31]. However, the neurocognitive mechanisms of these effects are poorly understood. Moreover, fMRI has not yet been used to assess how the brain changes after treatment in BN.

Using a naturalistic proof-of-concept, single-case design, we aimed to begin to address these gaps in the literature by examining changes in brain connectivity, inhibitory control task performance, and self-reported symptoms over treatment in a patient with severe BN and co-occurring emotional and behavioral dysregulation. We predicted that following the addition of lamotrigine to DBT, the patient would demonstrate new and increased neural connectivity in frontoparietal and frontolimbic networks that subserve behavioral and affective control, and consistent with past work in related samples [30, 32, 33], would report decreases in symptoms of affective and behavioral dysregulation. We also expected that the patient would show increases in task-based inhibitory control, indexed by go/no-go task performance, and increases in adaptive behaviors, indexed by DBT skills use.

## Materials and methods

### Patient

The patient^1^ was a single, 37-year-old, right-handed female with BN, who was admitted to the University of California, San Diego Eating Disorders Center for Treatment and Research 10-h partial hospitalization program after non-response to outpatient treatment. At admission, her body mass index was 20.2 kg/m^2^, and she was engaging in episodes of binge eating and purging via self-induced vomiting five times daily, restricting food intake outside of binge episodes, and exercising compulsively up to two hours daily. As such, she met the criteria for the “extreme” BN severity specifier in *DSM-5* [34], assessed by the Structured Clinical Interview for *DSM-5*, Research Version (SCID-5; [35]) Eating Disorders Module. Consistent with higher rates of comorbid mood, anxiety, and trauma-related disorders in “multi-impulsive BN” [13, 15], the patient also met *DSM-5* diagnostic criteria for Recurrent Major Depressive Disorder (MDD), Social Anxiety Disorder (SAD), and Post-Traumatic Stress Disorder (PTSD) per the Mini-International Neuropsychiatric Interview (MINI; Version 7.0; [36]). The patient reported a 26-year history of bulimic symptoms, and a long history of non-eating-related impulsive and emotionally dysregulated behaviors. This included parasuicidal behavior, a previous suicide attempt, difficulty managing anger, long-standing risky sexual behaviors, unstable and volatile relationships, and cannabis use at variable frequency. The patient received a score of 8 (out of 18) on the Borderline Personality Disorder module of the Structured Clinical Interview for *DSM-5* Personality Disorders (SCID-5-PD; [37]), suggesting BPD traits not meeting threshold for full criteria for the disorder (i.e., a score of 10 out of 18). Her IQ, as estimated by the Wechsler Test of Adult Reading (standard score = 116) was consistent with above-average intelligence [38].

### Assessment schedule

At admission, semi-structured diagnostic interviews were conducted by a trained bachelor’s-level research coordinator and height and weight were measured by a registered nurse in the clinical program. The patient completed biweekly computerized self-report assessments throughout the course of treatment (see Supplement for further detail regarding self-report measures and administration of psychodiagnostic assessments).

**Fig. 1:**
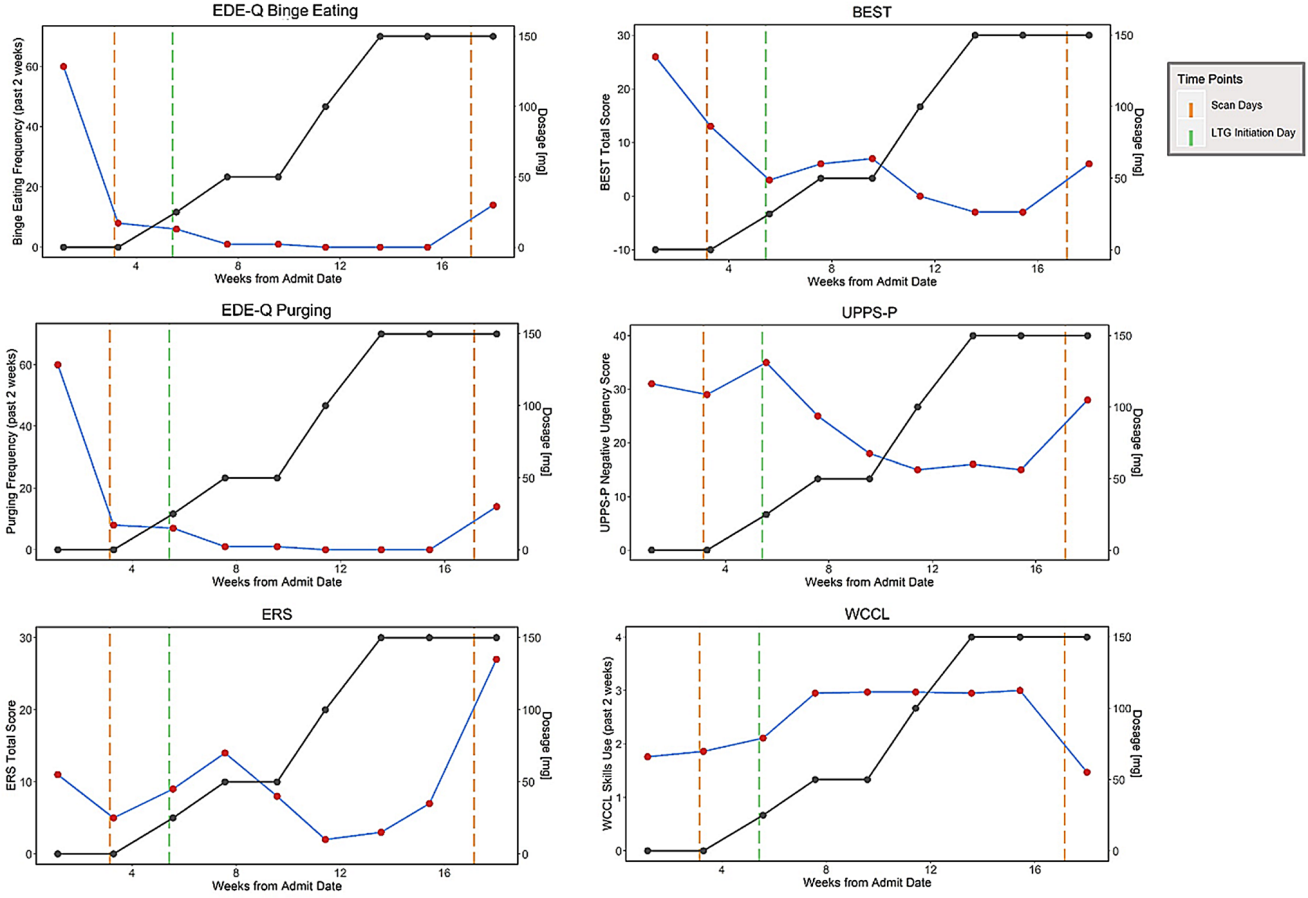
Self-reported symptom change over the course of treatment. Symptom change is represented by red dots and blue lines; lamotrigine dose change is represented by black dots and lines. *EDE-Q* Eating Disorder Examination Questionnaire, *ERS *Emotion Reactivity Scale, *BEST *Borderline Evaluation of Severity Over Time, *UPPS-P *UPPS Impulsivity Scale, Negative Urgency Subscale; *WCCL *ways of coping checklist skills use, *LTG* lamotrigine

Resting-state functional magnetic resonance imaging (rs-fMRI) scans and go/no-go task data were acquired 3 h after completing staff-monitored lunch in the treatment program at two-time points: T0 (DBT without lamotrigine), which took place 22 days after PHP admission/DBT initiation and 16 days before lamotrigine initiation); and T1 (DBT plus lamotrigine), which took place 25 days after a stable therapeutic dose of lamotrigine had been reached (see Fig. 1).

This patient was receiving contraceptive injections every three months; the T0 scan took place 41 days after her reported last menstrual period and her T1 scan took place 139 days after her reported last menstrual period. This study was approved by the UCSD Human Research Protections Program, and the patient provided written informed consent to participate.

### Neuroimaging

A high-resolution T1-weighted structural scan, a 5-min, T2*-weighted resting-state scan, and field maps were acquired on a 3T GE MR750 scanner using an eight-channel head-coil at the University of California, San Diego Keck Center for Functional Magnetic Resonance Imaging. See Supplemental Material for additional details regarding fMRI acquisition and preprocessing.

#### Regions of Interest

As the major targets of DBT and lamotrigine are affective and behavioral dysregulation, and prior research has documented neural changes after DBT and lamotrigine in the neural networks that support affective and behavioral control, we focused our analyses on frontoparietal and frontolimbic networks.

A frontoparietal control network was defined by eleven, 10-mm spherical regions of interest (ROIs) used in a previous study of attention-deficit/hyperactivity disorder that applied the directed connectivity approach described below (Table S1; [39]). These regions are integrally involved in both attentional and behavioral control, and have shown both structural and functional alterations in studies of adults and adolescents with BN [22, 40, 41].

An emotion regulation network was defined by fourteen, 5-mm spherical ROIs used in a previous study of emotion regulation network connectivity (Table S1; [42]). These regions were defined in a two-step procedure: a meta-analysis for the term “emotion regulation” via Neurosynth.org, and selection of seeds within these regions based on a neural model of emotion regulation [43]. BOLD time series were extracted from each ROI for individual-level network characterization at T0 and T1.

#### Directed Connectivity fMRI Analysis

Directed paths between all ROIs within each network were derived using Group Iterative Multiple Model Estimation (GIMME; [44]). Specifically, we used structural equation modeling via the indSEM function in the *gimme* package for R. This approach permits individual-level detection and quantification of connectivity among regions of the brain, controlling for all other network-wide influences, including each region’s lagged influence on itself. Directed paths between each ROI establish which ROI statistically predicts the other, both contemporaneously and lagged by one TR. This function conducts a model search and estimates individual-level paths to generate unique structure and estimates for each individual scan (see Supplemental Material for additional detail). We applied this function to scans from T0 and T1 to characterize structural equation models before and after treatment within each network.

### Go/no-go task

At T0 and T1, the patient completed a version of the go/no-go paradigm that has been used to assess neural correlates of inhibitory control in healthy individuals and clinical populations [45–47]. The design controls for the infrequency of no-go trials (a potential confound) with “oddball” go trials. All stimuli appeared on the screen for 500 ms with a jittered inter-stimulus interval (ISI; 1.6–2.0 s, mean ISI = 1.8 s). The patient was instructed to press the left or right button according to the direction of the stimulus arrow presented. A total of 11% of trials were no-go trials with upward-pointing arrows. On these trials, the patient was instructed to inhibit responding. Oddball go trials (arrows pointing either left or right at an upward, 45° angle) were also presented 11% of the time. A total of 416 trials were presented (48 no-go stimuli and 48 oddball go stimuli) across two blocks with a break in between. To control for practice effects, these blocks were presented in opposite orders at T0 and at T1. Responses to no-go stimuli (commission errors) serve as a key measure of deficits in action restraint [48].

### Self-report measures

A battery of self-report measures assessed changes in affective dysregulation and eating- and non-eating-specific behavioral dysregulation. The Emotional Reactivity Scale (ERS; [49]) assessed various facets of emotion reactivity, including arousal/intensity of emotion, persistence of emotion, and sensitivity to experiencing emotion, and the UPPS-P, Negative Urgency Subscale [50] assessed impulsivity in response to negative emotional states. Three adapted items from the Eating Disorder Examination—Questionnaire (EDE-Q; [51]) assessed relevant eating disorder behaviors: item 14, which assesses the frequency of binge eating episodes, and items 16 and 17, which assess the frequency of self-induced vomiting and laxative use (added together to assess purging behaviors). Given our bi-weekly assessment protocol, we asked the patient to report on these behaviors over the past 14 days (i.e., since the last assessment), instead of over the past 28 days. The total score from the Borderline Evaluation of Severity over Time (BEST; [52]) assessed cognitive, affective, and behavioral symptoms of BPD. Finally, the DBT Ways of Coping Checklist, Skills Use Subscale (WCCL; [53]) measured engagement in adaptive skills use in stress-inducing situations.

### Treatments

#### Dialectical Behavior Therapy

The Adult Program at the UCSD Eating Disorders Center for Treatment and Research includes partial hospital (PHP) and intensive outpatient (IOP) programs, both of which are based on an adapted form of adherent DBT for eating disorders, described elsewhere [54]. As noted above, DBT uses a cognitive-behavioral approach to decrease impulsive action in individuals with pervasive affective dysregulation and to increase adaptive skill use for managing heightened emotionality. Adaptive skills include distress tolerance, emotion regulation, mindfulness, interpersonal effectiveness skills, and functional analysis of behavior. Patients receive training in the use of these skills across three different formats: individual therapy, group therapy, and phone coaching. Within the UCSD program, patients receive group skills training two times a week, individual therapy one time a week, and phone consultation as needed outside of program.

The patient remained in treatment and received DBT for 20 weeks.^2^ In addition to DBT, the patient participated in other traditional psychotherapy groups (e.g., cognitive-behavioral therapy, supportive processing) and received nutritional care throughout her admission.

#### Pharmacotherapy

The patient had entered the program on escitalopram at a low dose (10 mg/day), and trazodone 100 mg at bedtime as needed for sleep. As SSRIs are considered first-line treatment of BN, initial medication management consisted of titrating escitalopram to 20 mg/day by the fifth week of PHP treatment. Lamotrigine was added for non-response in week 6 of PHP treatment. Over 8 weeks, the lamotrigine dose was titrated to 150 mg/day, which appeared therapeutic. The patient-reported medication adherence, except for missing two doses of lamotrigine midway through treatment, as well as accidentally taking 40 mg/day of escitalopram during the seventh week of treatment.

## Results

### Symptom change over time

The patient completed nine biweekly assessments during her time in treatment. Overall, the patient’s eating disorder and affective and behavioral dysregulation scores decreased over the course of treatment (Table S2; Fig. 1). Following lamotrigine initiation, and as the dose was increased, bulimic symptoms and negative urgency decreased, while DBT skills use increased. After several weeks on lamotrigine, the patient reported benefiting much more from DBT skills, especially mindfulness, pros/cons of binge eating and purging, and interpersonal effectiveness skills. As lamotrigine was titrated gradually to 150 mg/day over 8 weeks, improvement was so pronounced that the patient was stable enough to engage in a successful course of evidence-based individual psychotherapy for PTSD, which began in week 12 of PHP (Figure S1). For over two months, she continued to deny any eating-disorder behaviors and reported much improved mood and awareness of and control over emotions.

Self-report measures indicated a resumption of binge eating and purging at the patient’s last assessment time point. Of note, per her report to her clinicians, the patient had no eating disorder behaviors until 6 days after her second fMRI scan (T1). This 5-day relapse of binge eating, purging, and increased affective and behavioral dysregulation occurred in the context of multiple interpersonal stressors and impending treatment discharge. This pattern is not uncommon, as individuals with eating disorders are particularly vulnerable to interpersonal stress [55], and the multiple stressors associated with treatment termination may be particularly exaggerated in patients with high levels of emotion dysregulation [56]. The patient declined to complete a discharge self-report battery at week 20, but she denied any BN symptoms during her final two weeks in the program.

### fMRI results

#### Frontoparietal Control Network

**Fig. 2:**
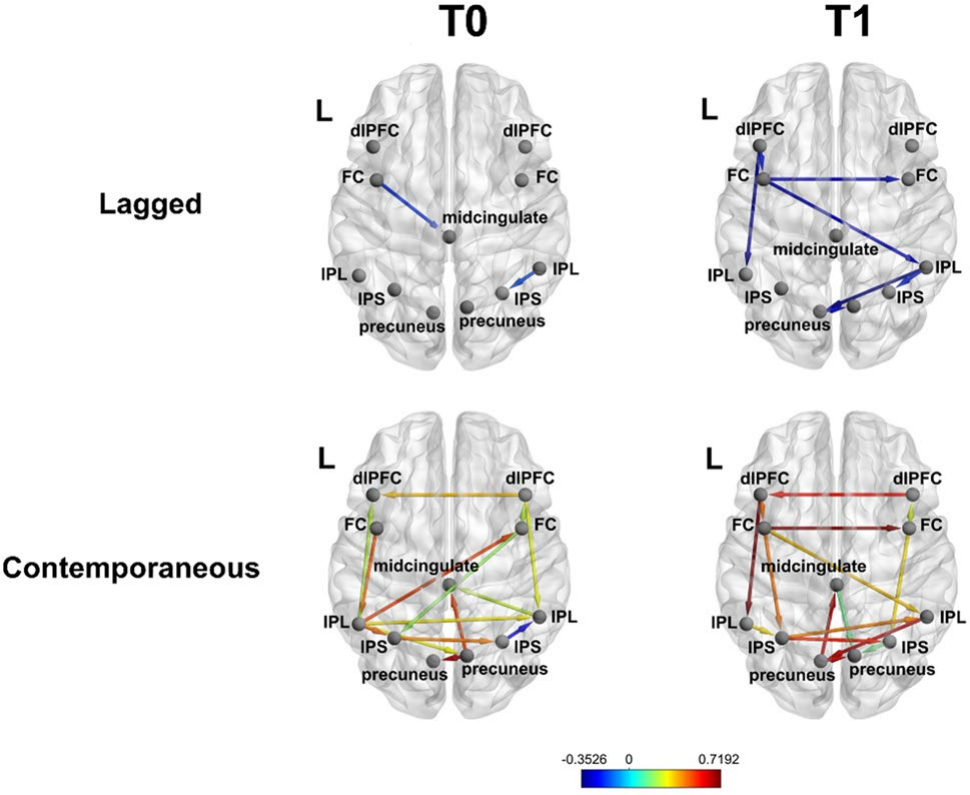
Effects of treatment on frontoparietal resting-state functional connectivity. Color represents the beta-weight strength of contemporaneous (bottom row) and lagged (top row) connections among frontoparietal regions at T0 (left column) and T1 (right column). Warm colors indicate positive beta weights, cool colors represent negative beta weights. *dlPFC *dorsolateral prefrontal cortex, *FC *frontal cortex, *IPL *inferior parietal lobule, *IPS *intraparietal sulcus, *L *left

Individual frontoparietal network connectivity is shown before initiation of lamotrigine (T0) and after reaching therapeutic dose (T1) in Fig. 2, with corresponding connectivity estimates in Figure S2. New, unilateral contemporaneous positive associations developed between intraparietal sulci (IPS) and frontal cortices (precentral gyri), and new contemporaneous positive connections between the left precuneus and the midcingulate, the left frontal cortex and left IPS, left frontal cortex and right inferior parietal lobule (IPL), and right IPL and left precuneus emerged. The connection between the left dlPFC and left IPL changed direction from T0 to T1, such that after treatment, the dlPFC showed a contemporaneous positive association with left IPL. In addition, several new lagged negative connections emerged between: left and right precuneus, left and right frontal cortex, between the left frontal cortex and right IPL, between left dorsolateral prefrontal cortex (dlPFC) and left frontal cortex and left IPL, and between right IPL and left precuneus.

#### Emotion Regulation Network

**Fig. 3:**
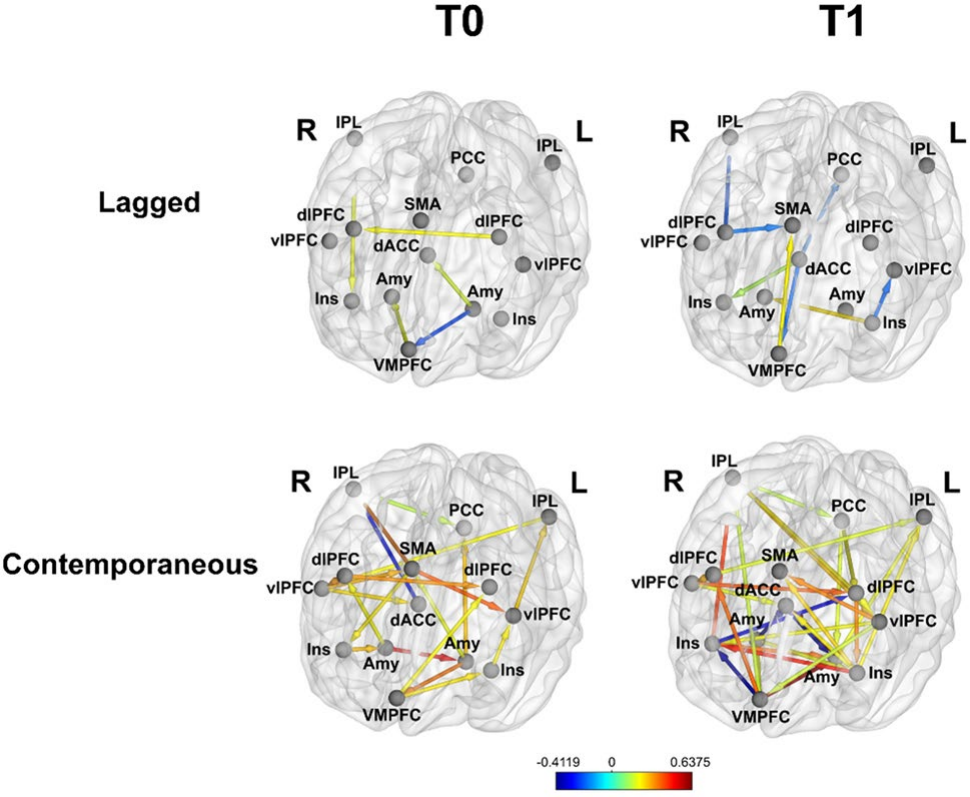
Effects of treatment on frontolimbic resting-state functional connectivity. Color represents the beta-weight strength of contemporaneous (bottom row) and lagged (top row) connections among frontolimbic regions at T0 (left column) and T1 (right column). Warm colors indicate positive beta weights, cool colors represent negative beta weights. *dlPFC *dorsolateral prefrontal cortex, *IPL *inferior parietal lobule, *Amy* amygdala,* Ins *insula,* dACC *dorsal anterior cingulate cortex, *PCC* posterior cingulate cortex, *SMA *supplementary motor area,* vlPFC *ventrolateral prefrontal cortex,* VMPFC *ventromedial prefrontal cortex,* R *right,* L *left

Individual frontolimbic network connectivity is shown before (T0) and after (T1) lamotrigine initiation in Fig. 3, with corresponding connectivity estimates in Figure S3. Several new positive connections emerged, including between the dorsal anterior cingulate cortex (dACC) and left amygdala. A previously lagged connection between the right IPL and the right insula became a strong, contemporaneous connection. New contemporaneous inverse associations also emerged between bilateral amygdalae and dACC, right insula and left dlPFC, and right insula and ventromedial prefrontal cortex (vmPFC). In addition, new lagged positive associations emerged from the vmPFC to supplementary motor area (SMA), dACC to right insula and left insula to right amygdala. New lagged inverse associations also emerged from left insula to left vlPFC, from dACC to vmPFC and to posterior cingulate cortex (PCC), and from right dlPFC to both SMA and right IPL.

### Go/no-go task performance

The patient’s frequency of errors on the go/no-go task decreased from T0 to T1 by almost 50% or more across all trial types (Figure S4A). This increase in accuracy was accompanied by slower reaction times across trial types (Figure S4B).

## Discussion

Relative to other psychopathologies, very little is known about brain-based changes associated with treatment in eating disorders. In this naturalistic, proof-of-concept study, we used fMRI, neurocognitive, and self-report assessments to track neural, cognitive, and behavioral changes in a patient with *DSM-5* BN who endorsed significant affective and behavioral dysregulation and received a course of DBT and lamotrigine. As expected, the patient showed increased and stronger resting-state functional connectivity within frontoparietal and frontolimbic networks and improved performance on a go/no-go task following the initiation of lamotrigine. Consistent with prior studies focused on symptom change [30, 31], the patient reported marked decreases in eating disorder behaviors and cognitive and behavioral dysregulation, many of which occurred more than six weeks into PHP treatment and coincided with the addition of lamotrigine. She also self-reported some positive changes in skills use. Enhanced cognitive and behavioral control may have contributed to the patient’s clinical improvements.

Rs-fMRI data suggest that increased skill use and symptom improvements were accompanied by increased frontoparietal connectivity, particularly between the left dlPFC and left IPL. These key regions of the executive control network are critical for introspective aspects of cognitive control, including self-directed attention, planning, and mentalizing, and are part of a frontoparietal subnetwork thought to contribute to emotional awareness and the regulation of thoughts and affect [57]. The dlPFC and IPL have been linked specifically to bulimic symptoms, as binge-eating frequency is inversely associated with cortical thickness in left dlPFC and right IPL in adolescents and adults with BN [22]. In addition, a recent meta-analysis implicated the dlPFC and parietal cortices, along with the SMA, supramarginal gyrus, midcingulate, and precuneus in dietary self-control [58]. Therefore, increased frontoparietal connectivity may have promoted enhanced executive control and reduced bulimic symptoms in this patient.

Within the emotion regulation network, new contemporaneous inverse associations emerged between prefrontal areas, particularly the dACC, and the amygdalae and insular cortices. At T1, this patient also showed new and increased connectivity between the left amygdala and the IPL and prefrontal cortices. Healthy individuals with higher reported self-control show increased connectivity during negative emotion regulation between left amygdala, the IPL, precuneus, prefrontal cortex, and supramarginal gyrus [59]. Thus, the regions between which this patient showed increased connectivity at rest may have played a role in her reported improvements in affect regulation.

Our results align with prior work focused on the separate effects of DBT and lamotrigine on the brain. Consistent with our findings of increased connectivity between dlPFC and IPL and between the dACC and amygdalae and insula following DBT, women with BPD (roughly one-third of whom had a comorbid eating disorder), who completed a 12-week course of DBT showed increases in grey matter volume in the IPL and ACC [60]. Few prior studies have focused on lamotrigine’s neurobiological effects in humans, but existing data suggest that the medication acutely increases resting-state connectivity among prefrontal regions in healthy males [61], and, after 8–12 weeks of lamotrigine, individuals with bipolar disorder show increased activation in prefrontal regions and decreased activation in amygdala when viewing emotional pictures [32, 33]. Our single-case data further support the notion that the combination of DBT and lamotrigine may be helpful for individuals with severe BN and elevated behavioral and cognitive dysregulation because of its effects on frontoparietal and frontolimbic self-regulatory control circuits.

Data from the go/no-go task suggest increased response inhibition from T0 to T1. Commission errors were reduced by almost 50%. Although these data are from only one patient, this improvement is greater than average commission error rate reductions seen after other treatments (e.g., approximate 23% reductions in even the most improved self-harming patients with BPD after 7 months of DBT [62]; 12% reductions following methyphenidate treatment for ADHD [63]). Because T0 and T1 were nearly 3.25 months apart and task block order was reversed at T1, it is unlikely, though possible, that these improvements are better explained by practice effects. In addition, RT increased across all trial types, but most notably during commission errors, which occurred less frequently at T1. These behavioral data may provide neurocognitive support for the patient’s report that she was more able to “slow down” to use therapeutic skills rather than act impulsively.

## Limitations

This is the first case report in the literature to assess a novel intervention’s effects on symptoms, neural activation, and behavioral task performance in BN. However, limitations should be acknowledged. We did not measure serum levels of medication, and this patient was also taking other psychotropic medications. Her dose of escitalopram changed during the course of treatment, which may have influenced results. However, symptom reductions continued or, in some cases, began after the patient’s lamotrigine dose was increased (Fig. 1), suggesting that it is less likely that all of our findings are attributable to SSRI treatment. Second, although data from this single-subject A-B design suggest that lamotrigine, above and beyond concurrent DBT, improved this patient’s symptoms, given our lack of a formal control or introduction of a second baseline, we cannot determine whether the changes observed are specifically attributable to lamotrigine, DBT, their combination, PTSD treatment, or other non-specific therapeutic factors (e.g., the structured environment of PHP/IOP level of care). Third, although prior results indicate that resting-state networks are stable within a 4-min time-series [64], and our scan was 5 min, longer resting-state scans may yield improved and more reliable connectivity estimates. Fourth, as the current fMRI and cognitive task data were collected from only one patient, the results should be interpreted with caution, and replication in a larger sample will be important to determine the reliability of our findings. Finally, we cannot rule out the possibility that reductions in binge eating and purging may have caused rather than been a consequence of neurocognitive changes; however, as the first scan was acquired after a significant initial reduction in these behaviors, this explanation of our findings may be less likely. Randomized, controlled designs including large samples, neuroimaging, and a variety of behavioral tasks and self-reports to measure self-regulation are needed to more clearly define the individual-level, mechanistic effects of these initially promising treatment approaches.

## Conclusions

This case report preliminarily supports the theory that DBT plus lamotrigine targets symptoms in patients with binge eating, purging, other impulsive behaviors, and significant affective lability by increasing inhibitory control and increasing connectivity among regions implicated in affective and behavioral self-regulation. In addition, our findings further highlight the potential promise of the GIMME approach for understanding neural alterations in individuals with eating disorders (65), and indicate that larger, systematic investigations of these post-treatment changes are warranted.

## What is already known on this subject?

Existing evidence-based treatments are less efficacious for individuals with multi-impulsive BN. Lamotrigine may provide benefit for these patients, particularly when combined with DBT.

## What this study adds?

Results provide initial support for the hypothesis that lamotrigine plus DBT targets symptoms in multi-impulsive BN by increasing frontoparietal and frontolimbic connectivity and enhancing inhibition.

## Supplementary Information

Below is the link to the electronic supplementary material.

Supplementary file1 (DOCX 1339 KB)

## Footnotes

1 Some details of patient characteristics were changed to disguise or remove any potentially identifying information. These alterations do not distort scientific meaning.

2 The patient began treatment at the PHP level (10 h/day, 6 days/week) and remained at this level of care for nine weeks. She stepped down to 6 h/day PHP for two weeks, and in week 12 of treatment, stepped down to 5-day IOP (4 h/day) for four weeks. At week 16, she stepped down to 3-day IOP for the remaining five weeks of treatment.

## References

[1] Arcelus J, Mitchell A, Wales J, Nielsen S (2011). Mortality rates in patients with anorexia nervosa and other eating disorders: a meta-analysis of 36 studies. Arch Gen Psychiatry.

[2] Fairburn CG, Cooper Z, Doll HA, Norman P, O'Connor M (2000). The natural course of bulimia nervosa and binge eating disorder in young women. Arch Gen Psychiatry.

[3] Golden N (2003). Eating disorders in adolescence and their sequelae. Best Pract Res Clin Obstet Gynaecol.

[4] Hudson J, Hiripi E, Pope H, Kessler R (2007). The prevalence and correlates of eating disorders in the National Comorbidity Survey Replication. Biol Psych.

[5] Mehler P, Crews C, Weiner K (2004). Bulimia: medical complications. J Womens Health.

[6] American Psychiatric Association (2000) Diagnostic & statistical manual of mental disorders: DSM: VI-TR, 4th edition. Association AP, editor, Washington

[7] Quadflieg N, Fichter MM (2019). Long-term outcome of inpatients with bulimia nervosa—Results from the Christina Barz Study. Int J Eat Disord.

[8] Linardon J, Wade TD (2018). How many individuals achieve symptom abstinence following psychological treatments for bulimia nervosa? A meta-analytic review. Int J Eat Disord.

[9] Mitchell J, Agras S, Wonderlich S (2007). Treatment of bulimia nervosa: Where are we and where are we going?. Int J Eat Disord.

[10] Rosval L, Steiger H, Bruce K, Israel M, Richardson J, Aubut M (2006). Impuslivity in women with eating disorders: problem of response inhibition, planning, or attention?. Int J Eat Disord.

[11] Gregorowski C, Seedat S, Jordaan G (2013). A clinical approach to the assessment and management of co-morbid eating disorders and substance use disorders. BMC Psychiatry.

[12] Harrop E, Marlatt G (2010). The comorbidity of substance use disorders and eating disorders in women: prevalence, etiology, and treatment. Addict Behav.

[13] Fichter MM, Quadflieg N, Rief W (1994). Course of multi-impulsive bulimia. Psychol Med.

[14] Lacey J, Evans C (1986). The impulsivist: a multi-impulsive personality disorder. Br J of Addiction.

[15] Myers TC, Wonderlich SA, Crosby R, Mitchell JE, Steffen KJ, Smyth J (2006). Is multi-impulsive bulimia a distinct type of bulimia nervosa: Psychopathology and EMA findings. Int J Eat Disord.

[16] Halmi K (2013). Perplexities of treatment resistance in eating disorders. BMC Psychiatry.

[17] Wilson G, Grilo C, Vitousek K (2007). Psychological treatment of eating disorders. Am Psychologist.

[18] Combs JL, Smith GT, Simmons JR (2011). Distinctions between two expectancies in the prediction of maladaptive eating behavior. Personal Individ Differ.

[19] Anestis M, Peterson C, Wonderlich S, Bardone-Cone A, Klein M, Mitchell J (2009). Affective lability and impulsivity in a clinical sample of women with bulimia nervosa: the role of affect in severely dysregulated behavior. Int J Eat Disord.

[20] Wu M, Hartmann M, Skunde M, Herzog W, Friederich H (2013). Inhibitory control in bulimic-type eating disorders: a systematic review and meta-analysis. PLoS One..

[21] Marsh R, Maia TV, Peterson BS (2009). Functional disturbances within frontostriatal circuits across multiple childhood psychopathologies. Am J Psychiatry.

[22] Berner LA, Stefan M, Lee S, Wang Z, Terranova K, Attia E (2018). Altered cortical thickness and attentional deficits in adolescent girls and women with bulimia nervosa. J Psychiatry Neurosci.

[23] Cyr M, Kopala-Sibley DC, Lee S, Chen C, Stefan M, Fontaine M (2017). Reduced inferior and orbital frontal thickness in adolescent bulimia nervosa persists over two-year follow-up. J Am Acad Child Adolesc Psychiatry.

[24] Berner LA, Wang Z, Stefan M, Lee S, Huo Z, Cyr M (2019). Subcortical shape abnormalities in bulimia nervosa. Biolog Psychiatry Cogn Neurosci Neuroimag.

[25] Westwater ML, Seidlitz J, Diederen KMJ, Fischer S, Thompson JC (2018). Associations between cortical thickness, structural connectivity and severity of dimensional bulimia nervosa symptomatology. Psychiatry Res Neuroimag.

[26] Linehan M (2018) Cognitive-behavioral treatment of borderline personality disorder. Guilford Publications

[27] Linehan M, Comtois K, Murray A, Brown M, Gallop R, Heard H (2006). Two-year randomized trial+follow-up of Dialectical Behavior Therapy versus Treatment-by-Experts for suicidal behaviors and borderline personality disorder. Arch Gen Psychiatry.

[28] Telch CF, Agras WS, Linehan MM (2001). Dialectical behavior therapy for binge eating disorder. J Consult Clin Psychol.

[29] Chen EY, Matthews L, Allen C, Kuo JR, Linehan MM (2008). Dialectical behavior therapy for clients with binge-eating disorder or bulimia nervosa and borderline personality disorder. Int J Eat Disord.

[30] Trunko ME, Schwartz T, Marzola E, Klein A, Kaye W (2014). Lamotrigine use in patients with binge eating and purging, significant affect dysregulation, and poor impulse control. Int J Eat Disord.

[31] Trunko ME, Schwartz TA, Berner LA, Cusack A, Nakamura T, Bailer UF (2017). A pilot open series of lamotrigine in DBT-treated eating disorders characterized by significant affective dysregulation and poor impulse control. Borderline Personal Disord Emot Dysregul.

[32] Jogia J, Haldane M, Cobb A, Kumari V, Frangou S (2008). Pilot investigation of the changes in cortical activation during facial affect recognition with lamotrigine monotherapy in bipolar disorder. Br J Psychiatry.

[33] Chang K, Wagner C, Garrett A, Howe M, Reiss A (2008). A preliminary functional magnetic resonance imaging study of prefrontal-amygdalar activation changes in adolescents with bipolar depression treated with lamotrigine. Bipolar Disord.

[34] American Psychiatric Association (2013) Diagnostic and Statistical Manual of Mental Disorders: Fifth Edition (DSM-5). Washington. American Psychiatric Association

[35] First M, Williams J, Karg R, Spitzer R (2015) user’s guide for the structured clinical interview for DSM-5 disorders, research version (SCID-5-RV). Arlington, VA, American Psychiatric Association

[36] Sheehan DV, Lecrubier Y, Sheehan KH, Amorim P, Janavs J, Weiller E (1998). The Mini-International Neuropspychiatric Interview (M.I.N.I.): the development and validation of a structured diagnostic psychiatric interview for DSM-IV and ICD-10. J Clin Psychiatry..

[37] First M, Williams J, Benjamin L, Spitzer R (2015) User’s guide for the SCID-5-PD (Structured Clinical Interview for DSM-5 Personality Disorder). Arlington, VA. American Psychiatric Association

[38] Wechsler D (2001) Wechsler test of adult reading: WTAR: Psychological Corporation

[39] Gates KM, Molenaar PCM, Iyer SP, Nigg JT, Fair DA (2014). Organizing heterogeneous samples using community detection of gimme-derived resting state functional networks. PLOS ONE..

[40] Marsh R, Horga G, Wang Z, Wang P, Klahr K, Berner L (2011). An FMRI study of self-regulatory control and conflict resolution in adolescents with bulimia nervosa. Am J Psychiatry.

[41] Marsh R, Stefan M, Bansal R, Hao X, Walsh BT, Peterson BS (2013). Anatomical characteristics of the cerebral surface in bulimia nervosa. Biol Psychiatry.

[42] Votinov M, Wagels L, Hoffstaedter F, Kellermann T, Goerlich KS, Eickhoff SB (2020). Effects of exogenous testosterone application on network connectivity within emotion regulation systems. Sci Rep.

[43] Etkin A, Buchel C, Gross JJ (2015). The neural bases of emotion regulation. Nat Rev Neurosci.

[44] Gates KM, Molenaar PC (2012). Group search algorithm recovers effective connectivity maps for individuals in homogeneous and heterogeneous samples. Neuroimage.

[45] Rubia K, Smith AB, Woolley J, Nosarti C, Heyman I, Taylor E (2006). Progressive increase of frontostriatal brain activation from childhood to adulthood during event-related tasks of cognitive control. Hum Brain Mapp.

[46] Schmidt A, Walter M, Gerber H, Schmid O, Smieskova R, Bendfeldt K (2013). Inferior frontal cortex modulation with an acute dose of heroin during cognitive control. Neuropsychopharmacology.

[47] Smith AB, Taylor E, Brammer M, Toone B, Rubia K (2006). Task-specific hypoactivation in prefrontal and temporoparietal brain regions during motor inhibition and task switching in medication-naive children and adolescents with attention deficit hyperactivity disorder. Am J Psychiatry.

[48] Swick D, Ashley V, Turken U (2011). Are the neural correlates of stopping and not going identical? Quantitative meta-analysis of two response inhibition tasks. Neuroimage.

[49] Nock M, Wedig M, Holmberg E, Hooley J (2008). The emotion reactivity scale: development, evaluation, and relation to self-injurious thoughts and behaviors. Behav Ther.

[50] Lynam D, Smith G, Whiteside S, Cyders M (2006). The UPPS-P: Assessing five personality pathways to impulsive behavior.

[51] Fairburn CG, Beglin SJ (2008) Eating disorder examination questionnaire (EDE-Q 6.0) Fairburn CG (Ed.), Cognitive behavior therapy and eating disorders. Guilford Press, New York. pp. 309–13

[52] Pfohl B, Blum N, St John D, McCormick B, Allen J, Black D (2009). Reliability and validity of the Borderline Evaluation of Severity Over Time (BEST): a self-rated scale to measure severity and change in persons with borderline personality disorder. J Pers Disord.

[53] Neacsiu A, Rizvi S, Vitaliano P, Lynch T, Linehan M (2010). The dialectical behavior therapy ways of coping checklist (DBT-WCCL): development and psychometric properties. J Clin Psychol.

[54] Brown T, Cusack A, Anderson L, Trim J, Nakamura T, Trunko M (2018). Efficacy of a partial hospital programme for adults with eating disorders. Eur Eat Disord Rev.

[55] Monteleone AM, Ruzzi V, Patriciello G, Cascino G, Pellegrino F, Vece A (2020). Emotional reactivity and eating disorder related attitudes in response to the trier social stress test: An experimental study in people with anorexia nervosa and with bulimia nervosa. J Affect Disord.

[56] McMain S, Korman L, Dimeff L (2001). Dialectical behavior therapy and the treatment of emotion dysregulation. J Clin Psychol.

[57] Dixon ML, De La Vega A, Mills C, Andrews-Hanna J, Spreng RN, Cole MW (2018). Heterogeneity within the frontoparietal control network and its relationship to the default and dorsal attention networks. Proc Natl Acad Sci.

[58] Han JE, Boachie N, Garcia-Garcia I, Michaud A, Dagher A (2018). Neural correlates of dietary self-control in healthy adults: A meta-analysis of functional brain imaging studies. Physiol Behav.

[59] Paschke LM, Dörfel D, Steimke R, Trempler I, Magrabi A, Ludwig VU (2016). Individual differences in self-reported self-control predict successful emotion regulation. Soc Cogn Affect Neurosci.

[60] Mancke F, Schmitt R, Winter D, Niedtfeld I, Herpertz SC, Schmahl C (2017). Assessing the marks of change: how psychotherapy alters the brain structure in women with borderline personality disorder. J Psychiatry Neurosci..

[61] Li X, Large CH, Ricci R, Taylor JJ, Nahas Z, Bohning DE (2011). Using interleaved transcranial magnetic stimulation/functional magnetic resonance imaging (fMRI) and dynamic causal modeling to understand the discrete circuit specific changes of medications: Lamotrigine and valproic acid changes in motor or prefrontal effective connectivity. Psychiatry Res Neuroimag.

[62] Ruocco AC, Rodrigo AH, McMain SF, Page-Gould E, Ayaz H, Links PS (2016). Predicting treatment outcomes from prefrontal cortex activation for self-harming patients with borderline personality disorder: a preliminary study. Front Hum Neurosci.

[63] Vaidya CJ, Austin G, Kirkorian G, Ridlehuber HW, Desmond JE, Glover GH (1998). Selective effects of methylphenidate in attention deficit hyperactivity disorder: A functional magnetic resonance study. Proc Natl Acad Sci.

[64] Van Dijk KR, Hedden T, Venkataraman A, Evans KC, Lazar SW, Buckner RL (2010). Intrinsic functional connectivity as a tool for human connectomics: theory, properties, and optimization. J Neurophysiol.

[65] Beltz AM, Moser JS, Zhu DC, Burt SA, Klump KL (2018). Using person-specific neural networks to characterize heterogeneity in eating disorders: Illustrative links between emotional eating and ovarian hormones. Int J Eat Disord.

