# Supplementary material for: Changes in cognitive and behavioral control after lamotrigine and intensive dialectical behavioral therapy for severe, multi-impulsive bulimia nervosa: an fMRI case study
Source: Eat Weight Disord. 2021 Oct 18;27(5):1919–28. doi: 10.1007/s40519-021-01308-z (PMC9122851; doi:10.1007/s40519-021-01308-z)
Supplement: Supplementary file 1 — Supplementary file1 (DOCX 1339 KB) [file 40519_2021_1308_MOESM1_ESM.docx]

**Supplemental Information**

**Materials and Methods**

**Clinical Interviews**

At treatment admission, the patient completed a battery of clinical interviews with good reliability and validity for assessing *DSM-5* psychiatric disorders and full-scale IQ. Interviews were conducted by trained bachelor’s-level research coordinator. The interviewer was supervised by the first author, a psychologist with extensive assessment experience, and diagnoses were determined via consensus. Prior to conducting interviews, the interviewer was required to complete an extensive training protocol and undergo several interviewing observations and feedback sessions by three psychologists. The interviewer was also required to attend 1 hour of weekly group supervision during which time they were required to present case diagnoses, supporting information for consensus, and answer questions posed by the consensus group regarding consideration of differential diagnoses. The assessor also attended 1 hour of weekly individual supervision during which interviews were reviewed, and additional individual consultation was available as needed.

**The Structured Clinical Interview for DSM-5, Research Version (SCID-5; (1)): Eating Disorders Module.** The SCID-5 is a semi-structured interview that has consistently demonstrated good reliability and validity (1). We used the SCID-5 Eating Disorders Module to assess the patient’s eating disorder diagnosis.

**The Structured Clinical Interview for DSM-5 Personality Disorders (SCID-5-PD (2)): Borderline Personality Disorder Module.** The SCID-5-PD is a semi-structured diagnostic interview used to assess DSM-5 personality disorders. For the current study, we used the BPD module to assess whether the patient met full DSM-5 criteria for BPD and to generate a total BPD symptom score.

**Mini-International Neuropsychiatric Interview (MINI; Version 7.0; (3)).** The MINI is a semi-structured interview that provides reliable and valid assessments of diagnoses according to criteria for the Diagnostic and Statistical Manual for Mental Disorders, 5^th^ edition (4, 5). We used the MINI to diagnose comorbid psychopathology (i.e., mood, anxiety, substance use, trauma-related, and obsessive-compulsive disorders).

**Wechsler Test of Adult Reading (WTAR).** At T0, the patient completed the WTAR to assess intelligence. The assessment consists of 50 English words with irregular pronunciations that participants are asked to read aloud. The measure was normed with the third edition of the Wechsler Adult Intelligence scale, and standard scores correlate highly with WAIS full-scale IQ (6).

**Self-Report Measures**

**Borderline Evaluation of Severity over Time (BEST; (7)).** The BEST is a 15-item self-report measure assessing symptoms commonly-observed in BPD. The scale has three subscales which gauge cognitive and affective symptoms of BPD (“Thoughts and Feelings”), behavioral symptoms of BPD [“Behaviors (Negative)”], and adaptive behaviors associated with self-regulation [“Behaviors (Positive)”]. Past work has supported the reliability and validity of the measure for assessing BPD symptom change over the course of treatment (7).

**Binge Eating and Purging.** Given our interest in assessing change in dysregulated eating behaviors characteristic of bulimia nervosa (i.e., binge eating and purging), we used three adapted items taken from the Eating Disorder Examination—Questionnaire (EDE-Q) (8) to assess these behaviors. Specifically, we used items assessing the frequency of binge eating episodes (#14) and purging behaviors (the sum of self-induced vomiting (#16) and laxative misuse (#17)). Prior studies in eating disorders (9), and specifically BN neuroimaging studies (10, 11), have used these behavioral frequency items as indicators of symptom severity and change in eating disorders. While the response format of the EDE-Q asks participants to report on their symptoms over the past 28 days, we adapted the time frame of these items to reflect our bi-weekly assessment protocol. Therefore, the patient was asked to report on the frequency of these behaviors in the past 14 days (i.e., since the last assessment).

**UPPS-P, Negative Urgency Subscale (12).** We used the UPPS-P, Negative Urgency Subscale to assess impulsivity in response to negative emotional states. The subscale has 12 items and has previously demonstrated good reliability and validity in a range of populations (12, 13); in the current study, we used the total score as an indicator of negative urgency.

**Emotional Reactivity Scale (ERS; (14)).** To measure potential changes in emotional reactivity, we used the ERS, which is a 21-item self-report measurement assessing various facets of emotion reactivity, including arousal/intensity of emotion, persistence of emotion, and sensitivity to experiencing emotion. The scale was developed in a sample of individuals who engage in non-suicidal self-injurious behavior and has demonstrated good reliability and validity (14).

**DBT** **Ways of Coping Checklist, Skills Use Subscale (WCCL; (15)).** The patient completed the 38-item DBT-WCCL, Skills Use Subscale to measure engagement in adaptive skills use in stress-inducing situations. Past work using the measure has indicated excellent psychometric properties (15).

**Neuroimaging**

Both scans and neurocognitive task data were acquired 3 hours after the patient completed a staff-monitored lunch in the treatment program (T0: a sandwich and soup; T1: a sandwich and chips).

**fMRI Acquisition.** Scans were acquired on a 3T GE MR750 scanner using an eight-channel head-coil at the University of California, San Diego Keck Center for Functional Magnetic Resonance Imaging. Structural data were acquired using a high-resolution T1-weighted MPRAGE sequence with prospective motion correction PROMO; (16) using ABCD study (17) parameters (TR = 2500ms; TI = 1060ms, TE = 2ms; orientation = sagittal; resolution = 1mm^3^, flip angle = 8°). Resting-state data were acquired during a 5-minute, eyes-open scan (T2*-weighted images; 3-mm interleaved slices with 0.5-mm spacing covering the whole brain; orientation = oblique, 15° up from the ACPC; TR = 1600ms; TE = 25ms; FOV= 19.2cm; flip angle = 76°; 64x64 matrix, 189 TRs). Field maps (TR = 1000ms; TE = 5.5ms and min full, FOV = 19.2cm; flip angle = 60°; 64x64 matrix) were collected to correct for geometric distortions. The patient was instructed to stay still and awake and look at a fixation-cross in the center of a projected display.

**fMRI Preprocessing.** Two steps were applied to minimize field inhomogeneity effects. First, raw EPI datasets were corrected for geometric distortions using fieldmaps via FSL (prelude and fugue tools to unwarp the images) and Analysis of Functional Neuroimages (AFNI) software (18). Next, we applied the N4 algorithm to remove the intensity non-uniformity bias field in Advanced Normalization Tools (ANTs). Subsequent pre-processing followed recommended steps in AFNI afni_proc.py and included voxelwise outlier rescaling and censoring (≥10% of the brain mask), slice time correction, nonlinear warping to the Montreal Neurological Institute 152 brain data set, motion correction that registers EPI volumes to that which has the minimum outlier fraction for motion, and spatial smoothing to 6-mm full-width half maximum. Time points with motion ≥0.2 mm or 0.2° (4.0% of data) were excluded from analyses.

**Directed Connectivity fMRI Analysis.** Input data for the GIMME algorithm is activation across the time series that has been extracted from each pre-specified spherical region of interest within a network. The resulting data vectors are subsequently treated as any other variable would be within a structural equation modeling framework. In other words, for each brain volume collected (i.e., for each TR), activation within each spherical region is approximated by the average of activation across all voxels within the region of interest, and these extracted values are subjected to subsequent analysis. The GIMME algorithm includes an automated procedure for determining the network paths that are statistically significant at the individual level. These are described in detail in (19). Briefly, when conducting analysis on a single subject’s scan, as we did in the current study, the algorithm calculates a modification index that indicates paths that would optimally improve the individual model if freed. The GIMME program identifies which modification indices are significant at the 0.01 level.

**Results**

**Table S1.** Coordinates of regions in frontoparietal control and emotion regulation networks

|  | x | y | z |
| --- | --- | --- | --- |
| Frontoparietal control |  |  |  |
| L dlPFC | -43 | 22 | 34 |
| R dlPFC | 43 | 22 | 34 |
| L FC | -41 | 3 | 36 |
| R FC | 41 | 3 | 36 |
| Midcingulate | 0 | -29 | 30 |
| L IPL | -51 | -51 | 36 |
| R IPL | 51 | -47 | 42 |
| L IPS | -31 | -59 | 42 |
| R IPS | 30 | -61 | 39 |
| L precuneus | -9 | -72 | 37 |
| R precuneus | 10 | -69 | 39 |
| Emotion regulation |  |  |  |
| L Amy | -18 | -4 | -16 |
| R Amy | 22 | -2 | -14 |
| L Ins | -38 | 20 | -4 |
| R Ins | 36 | 22 | -4 |
| dACC | -2 | 22 | 28 |
| PCC | -2 | -54 | 28 |
| R dlPFC | 34 | 26 | 40 |
| L dlPFC | -36 | 16 | 40 |
| SMA | 2 | 20 | 46 |
| R IPL | 52 | -48 | 44 |
| L IPL | -46 | -50 | 44 |
| R vlPFC | 48 | 18 | 26 |
| L vlPFC | -48 | 16 | 26 |
| vmPFC | 0 | 52 | -8 |

dlPFC = dorsolateral prefrontal cortex; FC = frontal cortex; IPL = inferior parietal lobule; IPS = intraparietal sulcus; Amy = amygdala; Ins = insula; dACC = dorsal anterior cingulate cortex; SMA = supplementary motor area; vlPFC = ventrolateral prefrontal cortex; vmPFC = ventromedial prefrontal cortex; R = right; L = left.

**Table S2.** Self-Report Assessments

| **Assessment** | **Week 1** | **Week 9** | **Week 18** | **Reference Values** |
| --- | --- | --- | --- | --- |
| BEST | 26.0 | 7.0 | 6.0 | In a sample of individuals presenting to treatment for BPD, the baseline mean BEST score was 41.5; SD = 10.7 (7). |
| Binge Eating (past 2 weeks) | 60.0 | 1.0 | 14.0 | In a sample of patients with BN, the median number of binge episodes reported in the past 28 days was 16.0 (20). |
| Purging (past 2 weeks) | 60.0 | 1.0 | 14.0 | In a sample of patients with BN, the median number of binge episodes reported in the past 28 days was 12.5 (20). |
| UPPS-P | 31.0 | 18.00 | 28.0 | In a recent study, healthy control women had a mean UPPS-P Negative Urgency score of 25.0; women with BN endorsed a mean score of 35.2 (21). |
| ERS | 11.0 | 8.0 | 27.0 | In the development sample for the ERS—a group of adolescents/young adults who engaged in self-injurious behavior—the mean ERS score was 36.7; SD = 17.5 (14); in a sample of female undergraduate participants, the mean of the measure was 29.9 SD = 18.7; (22). |
| WCCL | 1.8 | 3.0 | 1.5 | In a sample of women with BPD, the average pre-treatment WCCL Skills Use score was 1.5 (SD = 0.5); the average post-treatment skills use score was 1.9 (15). |

BN = Bulimia Nervosa; BEST = Borderline Evaluation of Severity Over Time; UPPS-P = UPPS Impulsivity Scale, Negative Urgency Subscale; ERS = Emotion Reactivity Scale; WCCL = Ways of Coping Checklist Skills Use

**
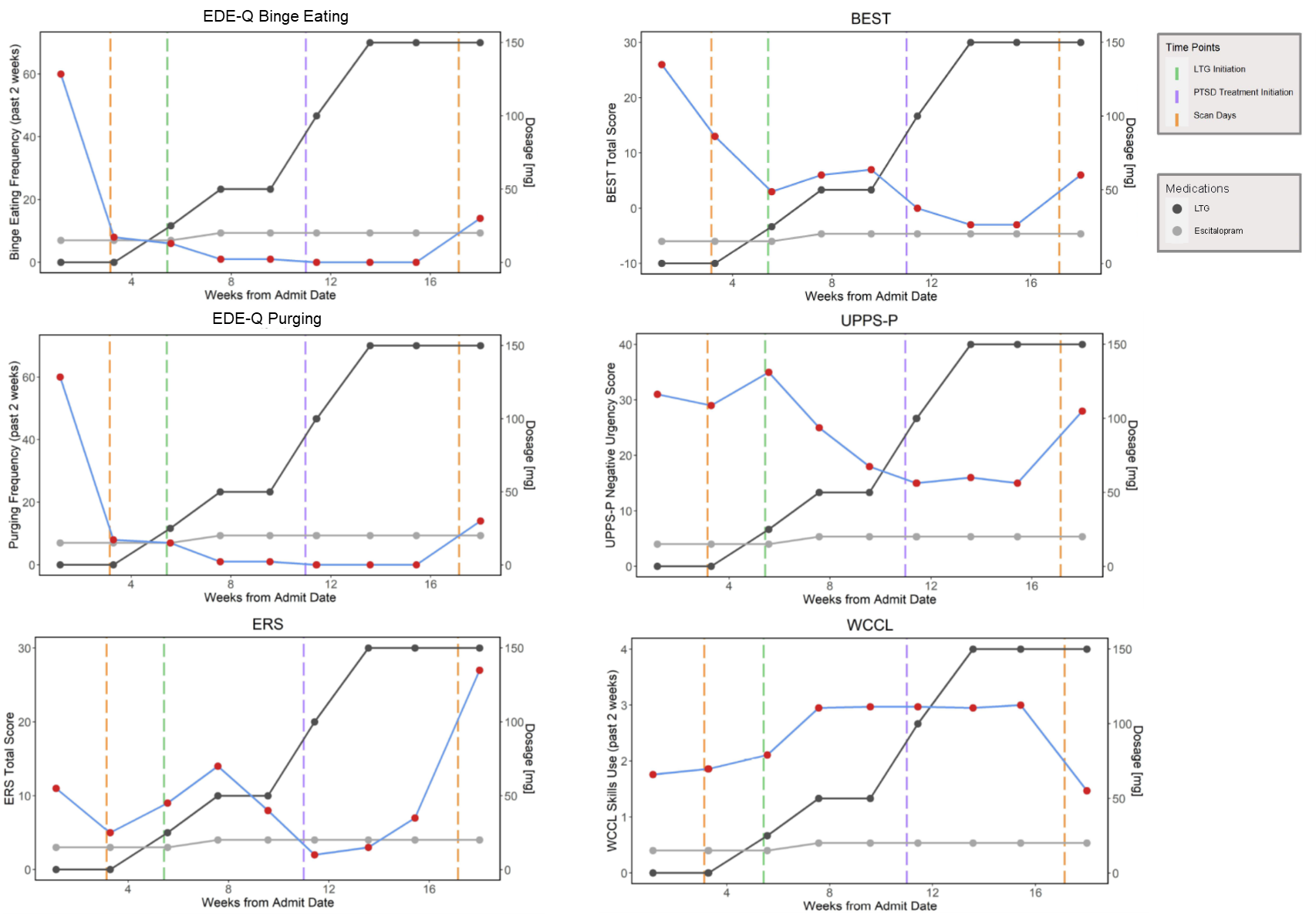
**

**Figure S1. Self-reported symptom change over the course of treatment.** Symptom change is represented by red dots and blue lines; lamotrigine dose change is represented by black dots and lines. Escitalopram doses is represented by grey dots and lines. The start of cognitive-behavioral PTSD treatment is indicated with a hatched purple line

**
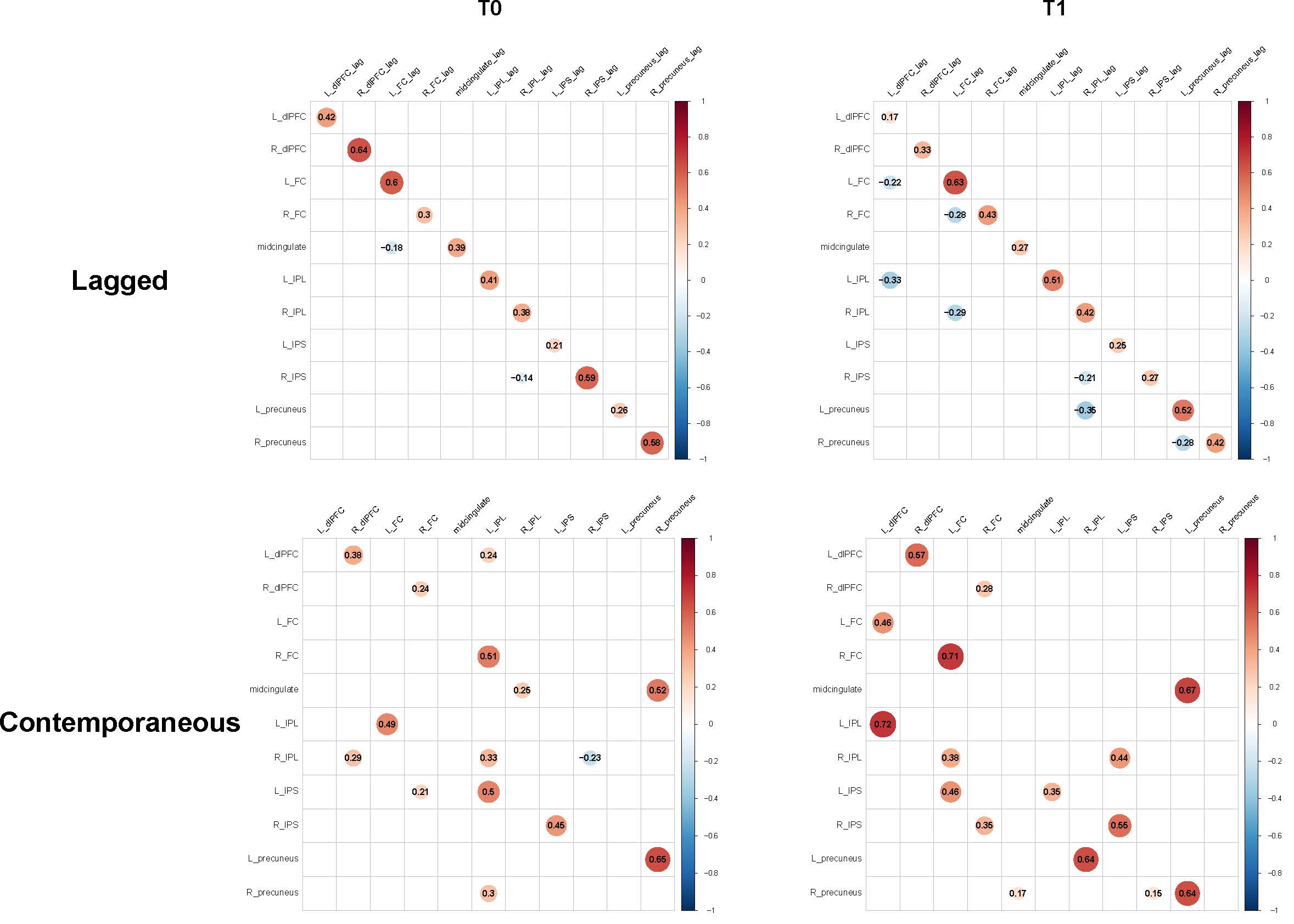
**

**Figure S2. Frontoparietal Beta Estimates of Lagged and Contemporaneous Associations at T0 and T1**

**
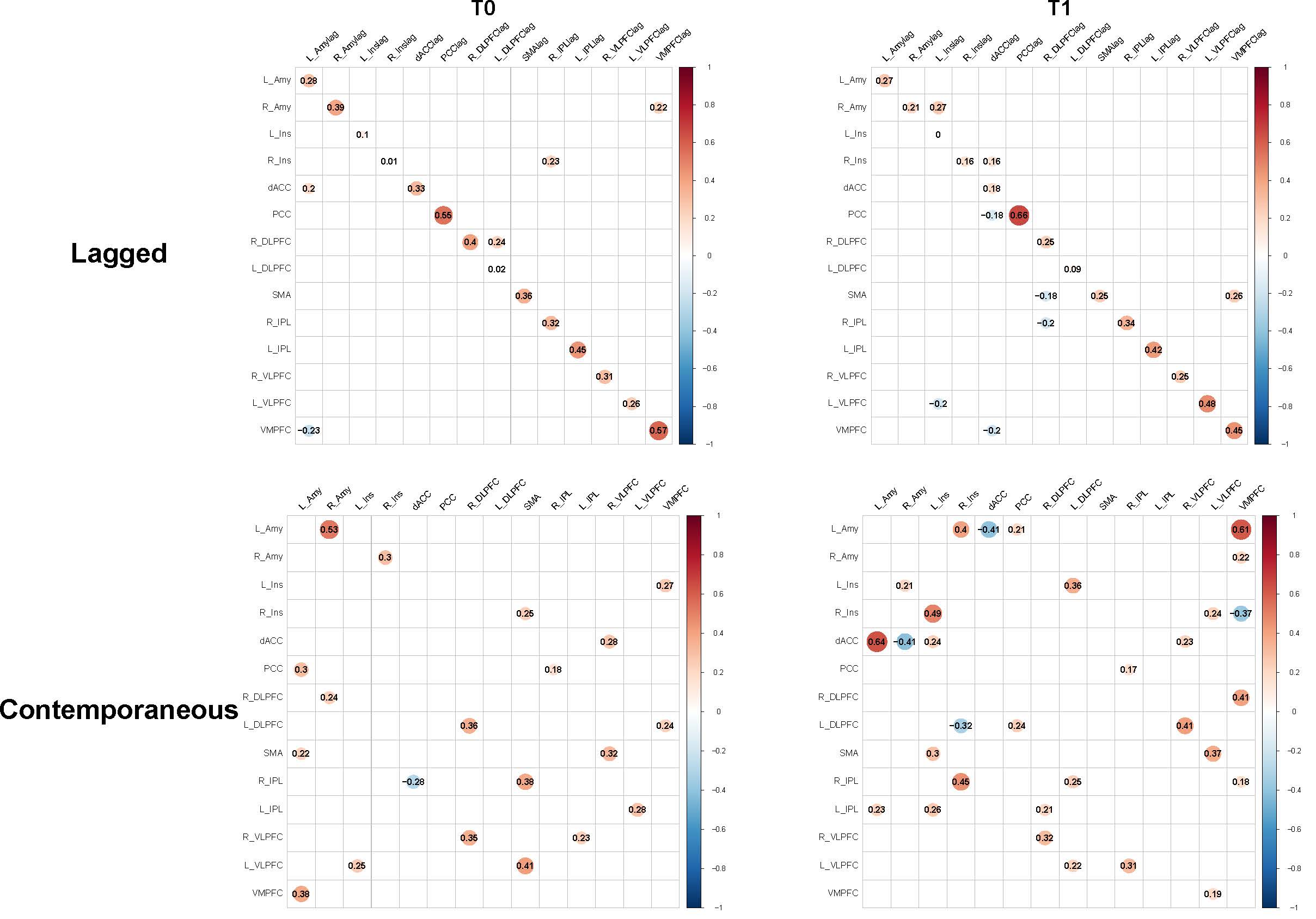
**

**Figure S3. Frontolimbic Beta Estimates of Lagged and Contemporaneous Associations at T0 and T1**

**
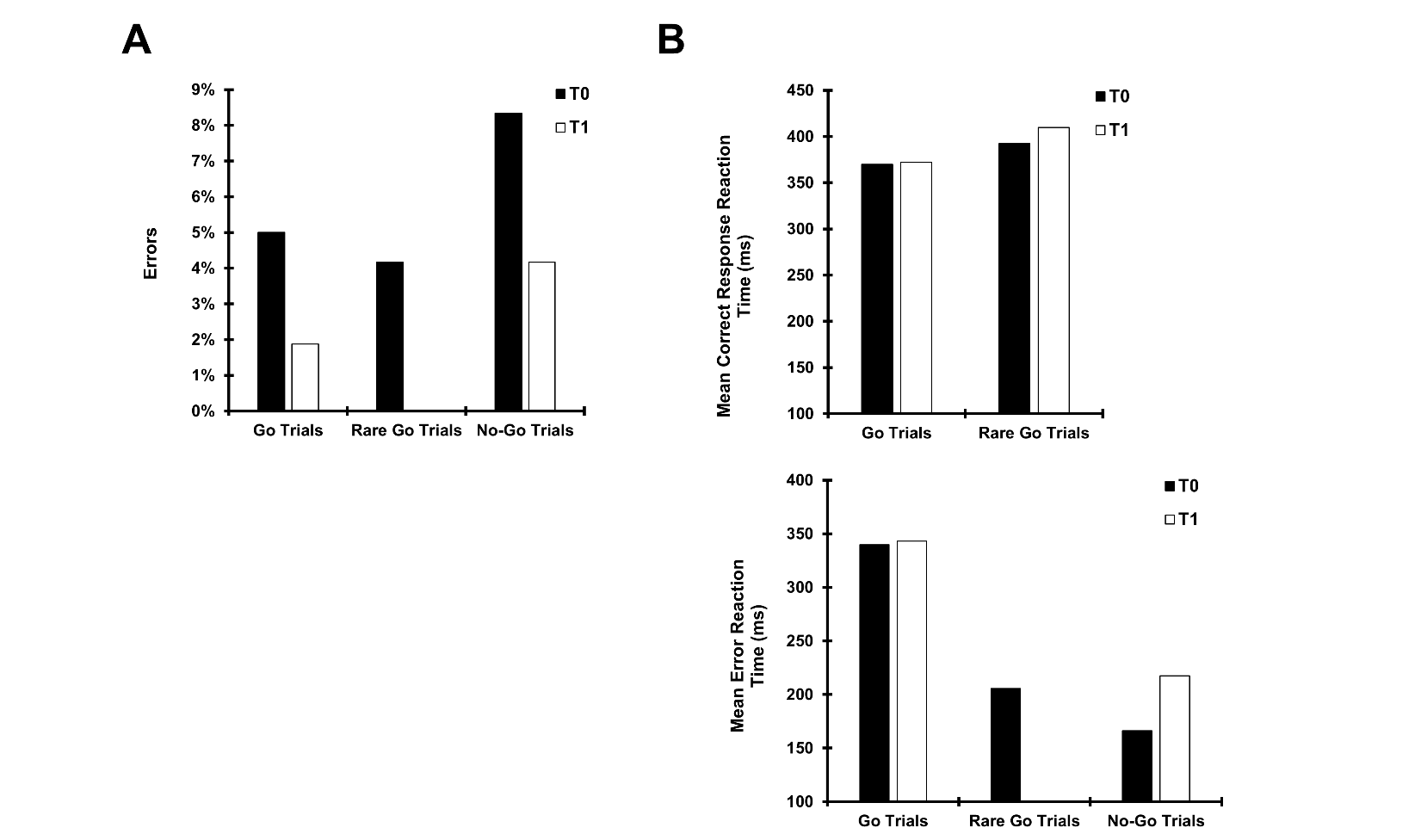
**

**Figure S4. Go/No-Go Task Performance.** A) Bars show the percentage of incorrect responses across trial types, both at T0 (black) and T1 (white). B) Bars represent the patient’s mean reaction times on each trial type at both time points. The patient made no rare go errors at T1.

**References**

1. First MB, Williams JBW, Karg RS, Spitzer R. Structured Clinical Interview for DSM-5—Research Version (SCID-5 for DSM-5, Research Version; SCID-5-RV). Arlington, VA, . American Psychiatric Association. 2015.

2. First M, Williams J, Benjamin L, Spitzer R. User’s Guide for the SCID-5-PD (Structured Clinical Interview for DSM-5 Personality Disorder). Arlington, VA, American Psychiatric Association. 2015.

3. Sheehan DV, Lecrubier Y, Sheehan KH, Amorim P, Janavs J, Weiller E, et al. The Mini-International Neuropspychiatric Interview (M.I.N.I.): the development and validation of a structured diagnostic psychiatric interview for DSM-IV and ICD-10. J Clin Psychiatry. 1998;59(20):22-33;quiz 4-57.

4. American Psychiatric Association. Diagnostic and Statistical Manual of Mental Disorders (DSM-V) 5th ed. Washington, DC American Psychiatric Association. 2013.

5. Sheehan DV, Lucrubier Y, Sheehan KH, Janaus J, Weiller E, Keskiner A, et al. The validity of the mini-international neuropsychiatric interview (MINI) according to the SCID-P and its reliability. Euro Psychiatry. 1997;12(5):232-41.

6. Strauss E, Sherman E, Spreen O. A compendium of neuropsychological tests: Administration, norms, and commentary. 3rd ed. Oxford University Press. . 2006.

7. Pfohl B, Blum N, St John D, McCormick B, Allen J, Black D. Reliability and validity of the Borderline Evaluation of Severity Over Time (BEST): a self-rated scale to measure severity and change in persons with borderline personality disorder. J Pers Disord. 2009;23(3):281-93.

8. Fairburn C, Cooper Z, O'Connor M. Eating disorder examination (16.0D). In : Fairburn CG (ed), *Cognitive Behavior Therapy and Eating Disorders*, 16th edition. New York: Guilford Press, pages 309-313. 2008.

9. Berg K, Peterson C, Frazier P, Crow S. Psychometric evaluation of the eating disorder examination and eating disorder examination-questionnaire: a systematic review of the literature. Int J Eat Disord. 2012;45(3):428-38.

10. Marsh R, Horga G, Wang Z, Wang P, Klahr K, Berner L, et al. An FMRI study of self-regulatory control and conflict resolution in adolescents with bulimia nervosa. Am J Psychiatry. 2011;168(11):1210-20.

11. Marsh R, Stefan M, Bansal R, Hao X, Walsh B, Peterson B. Anatomical characteristics of the cerebral surface in bulimia nervosa. Biol Pschiatry. 2015;77(7):616-23.

12. Lynam D, Smith G, Whitside SP, Cyders MA. The UPPS-P: Assessing five personality pathways to impulsive behavior. West Lafayette, IN: Purdue University. 2006.

13. Cyders M. Impulsivity and the sexes: Measurement and structural invariance of the UPPS-P Impulsive Behavior Scale. Assessment. 2013;20(1):86-97.

14. Nock M, Wedig M, Holmberg E, Hooley J. The emotion reactivity scale: development, evaluation, and relation to self-injurious thoughts and behaviors. Behav Therapy. 2008;39(2):107-16.

15. Neacsiu A, Rizvi S, Vitaliano P, Lynch T, Linehan MM. The Dialectical Behavior Therapy Ways of Coping Checklist (DBT-WCCL): Development and Psychometric Properties. J Clin Psychol. 2010;66(61):1-20.

16. White N, Roddey C, Shankaranarayanan E, Rettmann D, Santos J, Kuperman J, et al. PROMO: Real-time prospective motion correction in MRI using image-based tracking. Magn Reson Med. 2010;63(1):91-105.

17. Casey B, Cannonier T, Conley M, Cohen O, Barch D, Heitzeg M, et al. The Adolescent Brain Cognitive Development (ABCD) study: Imaging acquisition across 21 sites. Dev Cogn Neurosci. 2018;32:43-54.

18. Cox R. AFNI: software for analysis and visualization of functional magnetic resonance neuroimages. Comput Biomed Res. 1996;29:162-73.

19. Gates K, Molenaar P. Group search algorithm recovers effective connectivity maps for individuals in homogeneous and heterogeneous samples. Neuroimage. 2012;63(1):310-9.

20. Aardoom J, Dingemans A, Slof Op't Landt M, Van Furth E. Norms and discriminative validity of the Eating Disorder Examination Questionnaire (EDE-Q). Eat Behav. 2012;13(4):305-9.

21. Claes L, Islam M, Fagundo A, Jimenez-Murcia S, Granero R, Aguera Z, et al. The relationship between non-suicidal self-injury and the UPPS-P impulsivity facets in eating disorders and healthy controls. PLoS One. 2015;10(5):e0126083.

22. Kleiman E, Ammerman B, Look A, Berman M, McCloskey M. The role of emotion reactivity and gender in the relationship between psychopathology and self-injurious behavior. Pers Indiv Differ. 2014;69:150-5.
